# Natural Variation in the Promoter of *GmSPL9d* Affects Branch Number in Soybean

**DOI:** 10.3390/ijms25115991

**Published:** 2024-05-30

**Authors:** Duo Zhao, Haowei Zheng, Jiajia Li, Mingyue Wan, Kuo Shu, Wenhui Wang, Xiaoyu Hu, Yu Hu, Lijuan Qiu, Xiaobo Wang

**Affiliations:** 1 School of Agronomy, Anhui Agricultural University, Hefei 230036, China; zd19941213@163.com (D.Z.); zhenghaowei1219@163.com (H.Z.); lijia6862@163.com (J.L.); wmyalz@163.com (M.W.); 15056729246@163.com (K.S.); 18815755129@163.com (W.W.); huxiaoyu441@163.com (X.H.); huyu722227@163.com (Y.H.); 2 The National Key Facility for Crop Gene Resources and Genetic Improvement (NFCRI), Key Laboratory of Crop Gene Resource and Germplasm Enhancement (MOA), Institute of Crop Sciences, Chinese Academy of Agricultural Sciences, Beijing 100081, China

**Keywords:** soybean, branch number, *GmSPL9d*, nature variation, promoter activity

## Abstract

The branch number is a crucial factor that influences density tolerance and is closely associated with the yield of soybean. However, its molecular regulation mechanisms remain poorly understood. This study cloned a candidate gene *GmSPL9d* for regulating the soybean branch number based on the rice *OsSPL14* homologous gene. Meanwhile, the genetic diversity of the *GmSPL9d* was analyzed using 3599 resequencing data and identified 55 SNP/InDel variations, which were categorized into seven haplotypes. Evolutionary analysis classified these haplotypes into two groups: *GmSPL9d ^H-I^* and *GmSPL9d ^H-II^*. Soybean varieties carrying the *GmSPL9d ^H-II^* haplotype exhibited a significantly lower branch number compared with those carrying the *GmSPL9d ^H-I^* haplotype. Association analysis between the variation sites and branch number phenotypes revealed a significant correlation between the promoter variations and the branch number. Promoter activity assays demonstrated that the *GmSPL9d ^H-II^* promoter displayed significantly higher activity than the *GmSPL9d ^H-I^* promoter. Transgenic experiments confirmed that the plants that carried the *GmSPL9d ^H-II^* promoter exhibited a significantly lower branch number compared with those that carried the *GmSPL9d ^H-I^* promoter. These findings indicate that the variation in the *GmSPL9d* promoter affected its transcription level, leading to differences in the soybean branch number. This study provides valuable molecular targets for improving the soybean plant structure.

## 1. Introduction

Soybean is a crucial economic crop and a primary source of plant-based protein and oil for human consumption [1,2]. To meet the growing demand for soybean, it is essential to increase its yield. The yield of soybean is influenced by the plant architecture and planting density, with varieties that have a lower branch number typically demonstrating higher tolerance to dense planting [3,4]. Therefore, optimizing the plant structure is crucial for enhancing the soybean yield [5,6]. Only a few genes regulating soybean branch number have been identified, and genome-wide association analysis suggests that *GmBRC1* is a candidate gene for regulating the soybean branch number [7]. Natural variation in the *Dt2* promoter affects the transcription level of *GmAP1s*, significantly impacting the soybean branch number [8]. Thus, discovering genes that regulate soybean branching is vital for improving the density and yield.

*SQUAMOSA promoter-binding protein-like* (*SPL*) transcription factors form a major family of plant-specific transcription factors related to flower and vegetative development [9]. *IPA1* is a key regulatory factor for rice tiller formation and yield [10,11], and belongs to the *SPL* gene family. The *SPL* gene family was first identified in *Arabidopsis thaliana*, which possesses a highly conserved SBP domain containing 76 amino acids [12]. The *SPL* gene family plays a significant role in plant growth and development, including regulating branch formation, leaf primordium development, floral organ formation, and fruit ripening [13,14]. Among these, the *SPL* gene family has been extensively studied in the process of plant branch formation and development, such as the overexpression of *SPL10* weakening the apical dominance of *Arabidopsis* and promoting a higher branch number [15]. *SPL9* binds to *DELLA* to facilitate axillary bud formation in *Arabidopsis [16]*, and *SPL15* directly regulates the transcription level of *BRC1*, inhibiting *Arabidopsis* branch production [17]. The *miR156*-*SPL* module is an essential pathway that regulates soybean branch development, and *miR156b* regulates branch formation and development by cutting the transcript of *GmSPL9d* [18]. In recent years, the function of *GmSPL9d* in regulating the plant branch number has been reported [18,19], but its natural variation remains unexplored. Therefore, investigating the natural variation of the *GmSPL9d* gene is of significant importance.

This study utilized 3,599 soybean resequencing data to analyze the genetic diversity of the *GmSPL9d* gene. It was found that natural variations in the promoter region affected the soybean branch number. Transient transcriptional activity assays and GUS tissue staining experiments confirmed the impact of natural variations on promoter activity. Furthermore, transgenic *Arabidopsis* experiments demonstrated that natural variations in the promoter region influenced the branch number by affecting its transcriptional levels. The study also investigated the selection effects of different alleles of the *GmSPL9d* gene in different latitudinal regions, providing molecular targets for soybean plant structure improvement.

## 2. Results

### 2.1. Identification of Soybean SPL Gene Family Members

**Figure 1:**
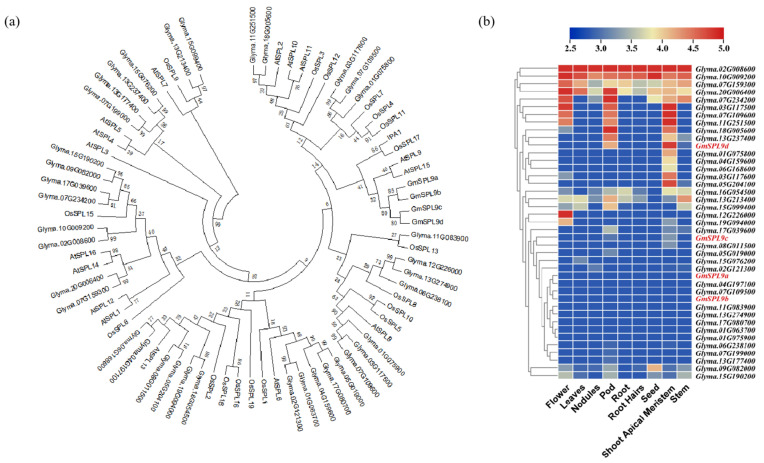
Phylogenetic and expression analyses of *GmSPLs.* (**a**) Phylogenetic tree of *SPLs* from *Arabidopsis*, soybean, and rice. (**b**) Expression analysis of *SPLS* in soybean, with the data from the Phytozome database.

To identify members of the *SPL* (*SQUAMOSA Promoter-Binding Protein-Like*) gene family, soybean genomic data were obtained from the Phytozome database. Utilizing the Pfam (PF03110) domain, 61 candidate *SPL* genes were initially identified. These candidates were further analyzed using the CDD, SMART, and Pfam databases to confirm the presence of the SBP domain, eliminate sequences lacking a complete SBP domain, and exclude distantly related sequences. Ultimately, 41 sequences with typical SBP domains were obtained. A phylogenetic tree was constructed using the selected *SPL* genes and previously reported rice and *Arabidopsis SPL* genes (Figure 1a and Figure S1a). The results show that *GmSPL9s* had the highest homology with the *IPA1* (*OsSPL14*) gene. Notably, *IPA1* plays a vital role in regulating plant architecture and yield, particularly in controlling the tiller number during the vegetative growth stage of rice [10,11]. Furthermore, the expression pattern of the soybean *SPL* family was analyzed, and it was found that *GmSPL9d* exhibited a higher expression in apical meristems (Figure 1b). Meanwhile, the expression pattern of *GmSPL9s* was further analyzed, and the results show that *GmSPL9d* was highly expressed in SAM due to soybean branches developing from SAM (Figure S1b). Therefore, *GmSPL9d* may play a crucial role in regulating the soybean branch number.

### 2.2. GmSPL9d Expression Pattern and Subcellular Localization

**Figure 2:**
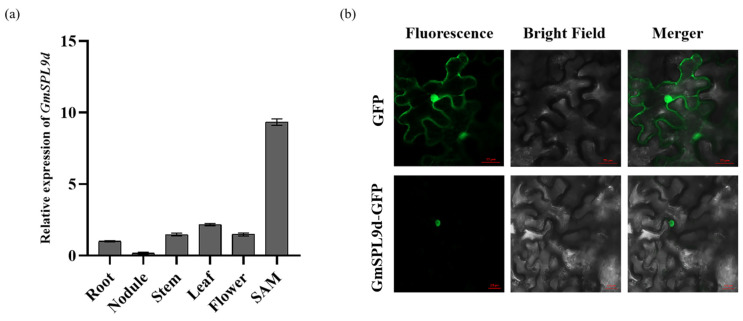
Expression patterns of *GmSPL9d* and the subcellular localization of *GmSPL9d.* (**a**) Gene expression of *GmSPL9d* in the root, nodule, stem, leaf, flower, and SAM. (**b**) *GmSPL9d* was localized to the nucleus. Scale bars, 20 μm.

Validation analysis of the expression pattern of *GmSPL9d* was conducted using qRT-PCR. The findings revealed that *GmSPL9d* exhibited a high expression in the shoot apical meristem (SAM) tissue (Figure 2a). To determine the subcellular localization of *GmSPL9d*, a GFP fusion protein was created by fusing *GmSPL9d* with GFP and subsequently expressed in *Nicotiana benthamiana* leaves. The results indicated that *GmSPL9d* was localized in the nucleus, which aligned with the characteristic localization of the *SPL* gene family transcription factors (Figure 2b).

### 2.3. Haplotype Analysis of GmSPL9d

**Figure 3:**
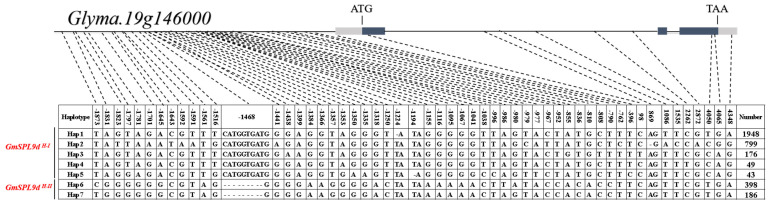
Haplotype analysis for *GmSPL9d* locus in the soybean population. Sequence and allelic variations in *GmSPL9d* locus, including 2 kb promoters and the full−length gene sequence. *GmSPL9d ^H-I^* (Hap 1−Hap 5) and *GmSPL9d ^H-II^* (Hap 6−Hap 7) represent seven different haplotypes detected by 55 polymorphic sites. The black boxes represent exons, the lines represent introns and the grey boxes represent UTR.

**Figure 4:**
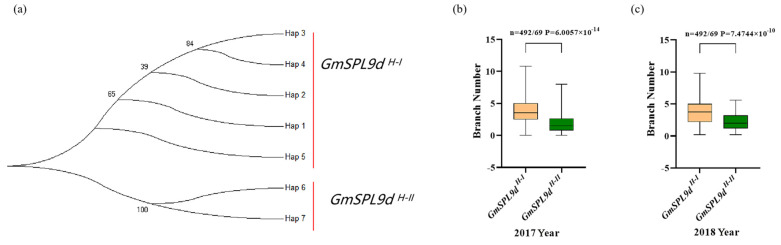
Branch number analysis of the different haplotypes in *GmSPL9d*. (**a**) Phylogenetic analysis of seven different haplotypes of *GmSPL9d*. (**b**,**c**) Boxplot of the branch number for two haplotypes in 2017 and 2018. Statistical significance (*P* = 6.0057 × 10^−14^ and *P* = 7.4744 × 10^−10^) of the difference between two haplotypes was determined by a two-sided Wilcoxon test. The center bold line represents the median; box edges indicate the upper and lower quantiles.

The genetic diversity of the *GmSPL9d* gene analysis results show that a total of 55 SNP/InDel variation sites were identified based on 3599 re-sequencing data (MAF > 0.05). These included 45 variations in the promoter region, 1 in the 5′ UTR, 1 in the 3′ UTR, 3 synonymous mutations, 5 variations in the intron, and a total of 7 haplotypes (Figure 3). Evolutionary analysis categorized the haplotypes into two types: *GmSPL9d ^H-I^* (including Hap 1, Hap 2, Hap 3, Hap 4, and Hap 5) and *GmSPL9d ^H-II^* (including Hap 6 and Hap 7) (Figure 4a). Through correlation analysis between the variation sites and branch number phenotype, it was observed that a significant number of variations in the promoter region were associated with the branch number (Table S2). Further analysis of the branch number phenotype of 561 materials revealed that the average branch numbers in 2017 and 2018 for the *GmSPL9d ^H-I^* types were 3.72 and 3.71, respectively, whereas for the *GmSPL9d ^H-II^* types, the average branch numbers in 2017 and 2018 were 1.95 and 2.25, respectively, representing a 43.5% decrease compared with *GmSPL9d ^H-I^* (Figure 4b,c). Meanwhile, we analyzed the frequency and geographical distribution of two haplotypes using resequencing data from 1037 landraces and 2267 elites. In the landraces, the main haplotype was *GmSPL9d ^H-I^* (93.5%), with a small percentage of *GmSPL9d ^H-II^* (6.5%). However, in the elites, the proportion of the *GmSPL9d ^H-II^* haplotype increased to 14.8% (Figure S2). Further analysis of the soybean varieties in China revealed a higher proportion of the *GmSPL9d ^H-II^* haplotype in high-latitude ecological regions (Figure S3). Previous studies demonstrated that *miR156b* regulates the branch number by targeting *GmSPL9d* (Sun et al., 2019); however, no variation was detected in the 20 bp base sequence at the target site (Figure S4). In conclusion, natural variations in the promoter region of *GmSPL9d* may represent an essential regulatory site for controlling the branch number in soybean.

### 2.4. The Activity of the GmSPL9d Promoter Affected the Branch Number in Soybean

**Figure 5:**
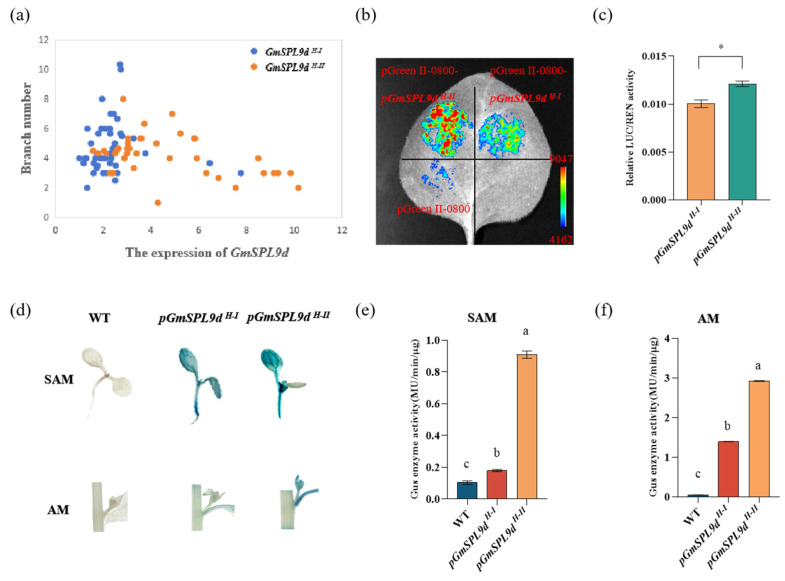
Promoter activity analysis of *pGmSPL9d ^H-I^* and *pGmSPL9d ^H-II^* in *GmSPL9d.* (**a**) Correlation analysis between the branch number and the *GmSPL9d* expression level in the AM (V4 stage) of 82 soybean accessions. (**b**,**c**) Promoter activity analysis of *GmSPL9d* using sequences 2000 bp upstream from the translation initiation site (*n*  =  3 biologically independent replicates, * indicates a significant difference at 0.05 level by Student’s *t*-test). (**d**) Different tissues (SAM and AM) of *GmSPL9d* transgenic arabidopsis staining results in *pGmSPL9d ^H-I^* and *pGmSPL9d ^H-II^*. (**e**,**f**) Comparison of different tissue (SAM and AM) GUS activities between *pGmSPL9d ^H-I^* and *pGmSPL9d ^H-II^*. Different letters indicate a significant difference in the GUS activity between *pGmSPL9d ^H-I^* and *pGmSPL9d ^H-II^* at the 0.05 level by a one-way ANOVA test (*n* = 3).

To investigate the mechanism by which *GmSPL9d* promoter variation influences the branch number, we initially performed a correlation analysis between the expression level of *GmSPL9d* in 82 natural germplasm AM tissues and the branch number phenotype. The results revealed a significant negative correlation between the expression level of *GmSPL9d* and the branch number (Figure S5). In addition, most *GmSPL9d ^H-I^* had a low expression and a high branch number, while most *GmSPL9d ^H-I^^I^* had a high expression and a low branch number (Figure 5a). Thus, it can be inferred that natural variation in the *GmSPL9d* promoter might regulate the branch number by impacting the gene’s expression level. To validate this hypothesis, we assessed the promoter activity of two haplotypes: *GmSPL9d ^H-I^* and *GmSPL9d ^H-II^*. The results demonstrated that the promoter activity of *GmSPL9d ^H-II^* was significantly higher than that of *GmSPL9d ^H-I^* (Figure 5b,c). Furthermore, a GUS histochemical staining experiment confirmed that the GUS enzyme activity in the SAM and AM tissues of *Arabidopsis thaliana* with the *GmSPL9d ^H-II^* promoter type was notably higher than that with the *GmSPL9d ^H-I^* promoter type (Figure 5d–f). These findings indicate that natural variation in the *GmSPL9d* promoter impacted the promoter activity, thereby influencing the branch number.

### 2.5. GmSPL9d ^H-II^ Haplotype Inhibited Arabidopsis Branching

**Figure 6:**
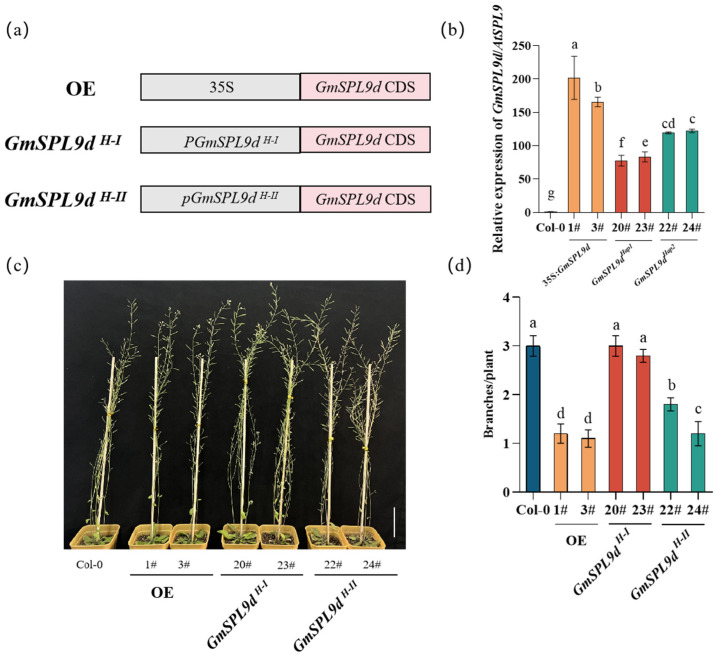
Effect of different *GmSPL9d* promoters on the branching of Arabidopsis. (**a**) Schematic diagram of different constructs using pCAMBIA1305 as the backbone vector, including 35S: *GmSPL9d*, *PGmSPL9d ^H-I^*: *GmSPL9d*, and *PGmSPL9d ^H-II^*: *GmSPL9d*. (**b**) qRT-PCR analysis of *GmSPL9d/AtSPL9* expression in the transgenic *Arabidopsis* lines (20 days of axillary meristem). (**c**) Transgenic phenotype in Col-0. Scale bar, 5 cm. (**d**) Quantitative analysis of the branch number in wild-type plants (Col-0) and transgenic plants (*n* = 10). The data were collected at 45 days after emergence from the soil. ACTIN7 was used as an internal control for gene expression. All data are represented as mean ± SE. Different letters represent a significantly difference (one-way ANOVA test; *p* < 0.05).

To further confirm the role of the two haplotypes in regulating the branch number, we transformed the CDS encoding region of *GmSPL9d* driven by 35S, *pGmSPL9d ^H-I^*, and *pGmSPL9d ^H-II^* promoters into Col-0 *Arabidopsis* (Figure 6a and Figure S6). The expression level of *GmSPL9d ^H-II^* transgenic lines in the axillary meristem was significantly higher than that of *GmSPL9d ^H-I^*, and both haplotypes exhibited significantly lower expression levels compared with the over-expression plants (Figure 6b). Consistently, the branch number in *GmSPL9d ^H-II^* was significantly lower than in *GmSPL9d ^H-I^*, and both haplotypes had a significantly higher branch number compared with the over-expression plants (Figure 6c,d). Notably, there was no significant difference in the branch numbers between the *GmSPL9d ^H-I^* and Col-0 plants. These results indicated that *GmSPL9d* could only inhibit *Arabidopsis* branching when expressed at a high level, and furthermore, the *GmSPL9d ^H-II^* haplotype exhibited a higher expression level, thereby suppressing *Arabidopsis* branching.

## 3. Discussion

Transcription factors known as *SPLs* have been extensively researched in plants and are known to play crucial roles in plant growth and development [13,20,21,22,23,24]. *SPLs* form a small gene family, with 16 *SPL* genes in *Arabidopsis* and 19 in rice [25]. In this study, we identified 41 *SPL* genes in soybean. Compared with *Arabidopsis* and rice, the number of soybean *SPL* genes is higher. The genome-wide duplication (WGD) events in soybean evolutionary history may explain this phenomenon. WGD is very common in plants, leading to double gene copies in the genome. The functional divergence of duplicate gene pairs is the source of new genes. Soybean has been reported to experience at least two WGD events, approximately 59 and 13 million years ago, resulting in a highly duplicated genome, with nearly 75% of the genes present in multiple copies [26]. Therefore, a greater expansion of *SPL* genes may have occurred in the soybean genome than in other species. It was further discovered that *GmSPL9s* (*GmSPL9a*, *GmSPL9b*, *GmSPL9c*, *GmSPL9d*) exhibits the highest homology with *IPA1* (*OsSPL14*). *IPA1*, also known as a “star gene” in rice, is a key regulator of tillering, stalk strength, and panicle traits (Jiao et al., 2010; Miura et al., 2010). Therefore, *GmSPL9s* may play an important role in the regulation of soybean plant phenotypes. As a member of the soybean *SPL* gene family, *GmSPL9d* is highly expressed in the shoot apical meristem (SAM), where the shoot apical meristem (SAM) contains undifferentiated stem cells that are responsible for the initiation of aboveground organs [27]. Thus, *GmSPL9d* may play a critical role in multiple traits. As expected, previous studies do validate this. For instance, Arbuscular mycorrhizal fungus Rhizophagus irregularis alleviates drought stress in soybean by overexpressing the *GmSPL9d* gene by promoting the photosynthetic apparatus and regulating the antioxidant system [28]. The *miR156b-GmSPL9d* module modulates nodulation by targeting multiple core nodulation genes in soybean [29]. In addition, the *miR156b-GmSPL9d* module also regulates the branch number in soybean [18]. Despite numerous studies that demonstrated the regulatory role of *GmSPL9d* in the soybean branch number, the impact of natural variation on gene expression and function remains unexplored [18,19]. Therefore, investigating the natural variation in *GmSPL9d* contributes to the expansion and enhancement of our understanding of the molecular mechanisms underlying the soybean branch number.

By conducting a genetic diversity analysis of *GmSPL9d*, two haplotypes, namely, *GmSPL9d ^H-I^* and *GmSPL9d ^H-II^*, were identified that exhibited significant differences in the branch number. In rice, a natural variation in the *IPA1* coding region (C-T) resulted in the inability of *miR156* to recognize and target it, leading to differences in the branch number [10,11]. In this study, no natural variation was found in the *miR156b* targeting sequence, and no non-synonymous mutations were detected in the coding region of *GmSPL9d*. However, a considerable number of SNP/InDel variation sites were discovered in the promoter region, which were significantly associated with the branch number. Increasing evidence suggests that the natural variations present in promoter regions also play critical roles in altering agronomic traits by regulating the gene expression levels [30,31]. For example, segmental deletion in the promoter region of qSW5/GW5 alters its expression level and results in slender grains [32,33]. The insertion of a 148/150 bp fragment in the *GmCHX1* promoter leads to differences in salt tolerance [34]. This suggests that natural variation in the promoter region of *GmSPL9d* may be the primary factor influencing the difference in the branch number between haplotypes.

The different haplotypes of *GmSPL9d* exhibited significant differences in the promoter activity. *GmSPL9d ^H-II^* showed a significantly higher promoter activity than *GmSPL9d ^H-I^* in both the transient and stable transformation assays. Importantly, significant differences in GUS enzyme activity driven by *GmSPL9d ^H-II^* and *GmSPL9d ^H-I^* promoters were observed in the shoot apical meristem (SAM) and axillary meristem (AM) of transgenic *Arabidopsis*. SAM and AM are crucial tissues involved in plant branching, as extensively reported [35,36,37,38]. Furthermore, when the *GmSPL9d* gene was expressed in *Arabidopsis* using different promoters, the overexpression of *GmSPL9d ^H-II^* resulted in a significantly lower branch number compared with *GmSPL9d ^H-I^* plants, indicating a synergistic relationship between the phenotype and expression level. Previous studies on salt tolerance traits in soybean demonstrated that the transcript levels of ProHap2: *GsERD15B* were significantly higher than 35S: *GsERD15B* and ProHap1: *GsERD15B*, indicating a stronger salt tolerance [39]. Similarly, in this study, the *GmSPL9d ^H-II^* promoter activity was significantly higher than that of *GmSPL9d ^H-I^*, and plants carrying the *GmSPL9d ^H-II^* promoter type exhibited significantly lower branch numbers compared with those carrying the *GmSPL9d ^H-I^* promoter type. Interestingly, the expression level of *GmSPL9d ^H-I^* was significantly higher than that of Col-0, but no difference in the branch number was observed. This is consistent with previous studies, where a significant reduction in the branch number in Col-0 plants was only observed at high expression levels of *GmSPL9d* [18]. It is possible that *GmSPL9d* is targeted by *miR156s* in *Arabidopsis*, and only higher expression levels can exert the corresponding gene function. These results suggest that natural variation in the promoter region of *GmSPL9d* affects the soybean branch number by influencing its expression level. We have not disclosed the causal genetic variations responsible for the functional divergence of the two haplotypes. A further investigation of the upstream regulatory genes may help us to determine which polymorphisms are essential for the transcription of *GmSPL9d*, which will make the regulatory network more complete.

## 4. Materials and Methods

### 4.1. Plant Material and Growth Conditions

A total of 561 soybean natural germplasm resources were employed as experimental materials. The experiment was conducted using a randomized block design with a row length of 2 m, row spacing of 40 cm, plant spacing of 10 cm, and comprising two row blocks. The soybean plants were sown at the experimental base of Anhui Agricultural University (Table S1). *Col-0 Arabidopsis thaliana* served as the transgenic receptor, while *Nicotiana benthamiana* was used as the transient transformation receptor. These plants were grown in a greenhouse under conditions of 22 °C temperature, 16 h of light, and 8 h of darkness. These planting conditions were selected to provide an appropriate environment that ensured the accuracy and reproducibility of the experiment.

### 4.2. RNA Extraction and qRT-PCR

Total RNA was isolated from soybean tissues using an RNA prep Pure Plant Kit (TaKaRa, Otsu, Japan), and first-strand cDNA was synthesized using a PrimeScript™ 1st Strand cDNA Synthesis Kit (TaKaRa). qRT-PCR analysis was performed with a CFX96TM Real-time System (BIO-RAD, Hercules, CA, USA) with 10 μL of SYBR Mix (VAZYME, Nanjing, China), 6 μL of ddH2O, 1 μL of each primer, and 2 μL of cDNA template to a final volume of 20 μL. The implemented reaction procedure was 95 °C for 60 s, followed by 40 cycles of 95 °C for 30 s, 60 °C for 30 s, and 72 °C for 30 s. The relative gene expression levels were calculated using the 2^−ΔΔCT^ method (Livak and Schmittgen. 2001) by normalization to GmACTIN (Glyma.19G147900) [40] or AtACTIN7 (AT5G09810) in soybean and Arabidopsis, respectively [41]. All the primers used in this experiment are listed in Table S3.

### 4.3. Identification and Bioinformatics Analysis of GmSPL9d

The latest reference genome of soybean and the hidden Markov model (HMM) profile of *SPL* (PF03110) were downloaded from the Phytozome soybean genome and PFAM databases. The hmmsearch program in HMMER software [42] was used to search the SPL HMM profile against the soybean genome, and the reliable results were screened based on an E-value less than or equal to 1 × 10^−10^. The soybean-specific SPL HMM profile was constructed using the hmmbuild program, and then the soybean reference genome was searched again with an E-value threshold 1 × 10^−10^. To avoid missing other members of the *SPL* gene family, the SPL-type protein sequences from soybean reported on the NCBI website were used as query sequences for BLASTP alignment with a E-value threshold of 1 × 10^−5^. We combined the genes predicted using the two methods and removed the duplicates. If multiple transcripts corresponding to a single SPL gene were obtained, only the most reliable one was retained. The protein sequences of the putative genes were submitted to the NCBI–CDD3 and PFAM online databases for verification, and the putative genes without SBP domains were excluded. The genes with an E-value greater than or equal to 1 × 10^−20^ predicted by the Pfam database were also excluded. Finally, members of the soybean SPL gene family were obtained with high confidence. For the multiple-alignment analysis, the amino acid sequences of SPL proteins from soybean, *Arabidopsis*, and rice were downloaded from the NCBI website. The phylogenetic tree was constructed with MEGA7.0 software using the NJ method [43]. The bootstrap tests were conducted with 1000 replicates, and other parameters were set to default.

### 4.4. Subcellular Localization

The coding sequence (CDS) of *GmSPL9d* was amplified from the soybean variety Williams82 using specific primers (Table S3). The CDS of *GmSPL9d* was then cloned into pCAMBIA1305-GFP using the homology recombination kit (VAZYME, Nanjing, China). The resulting construct was transformed into *Agrobacterium* GV3101 and subsequently injected into the leaves of *Nicotiana benthamiana*. After a culture period of 36–48 h, the subcellular localization of GmSPL9d within the epidermal cells of the inoculated tobacco leaves was observed using a laser scanning confocal microscope (Zeiss LSM880, Carl Zeiss, Jena, Germany).

### 4.5. Histochemical Analysis of GUS Activity

The 2000 bp sequence of the *GmSPL9d* promoter was downloaded from Phytozome v10 (https://phytozome.jgi.doe.gov/pz/portal.html, accessed on 13 December 2022 ). DNA fragments of the Baimao (*GmSPL9d ^H-I^*) and Heihe 37 (*GmSPL9d ^H-II^*) varieties were amplified and cloned into the phzm33 vector, followed by transformation into the GV3101 strain (Shanghai Weidi Biological, Shanghai, China). Transgenic *Arabidopsis* was obtained using the floral dip method. X-gluc (5-bromo-4-chloro-3-indolyl β-d-glucuronide) and GUS staining buffer (Jefferson et al., 1987) containing 1 mM X-gluc enzyme (Gold BioTechnology, St. Louis, MO, USA), 100 mM sodium phosphate (pH 7.5), 0.5 mM potassium ferrocyanide, 0.5 mM potassium ferricyanide, 10 mM EDTA, and 0.1% (*v*/*v*) Triton X-100 were used to stain the transgenic *Arabidopsis* seedling SAM and AM. All treatments were incubated at 37 °C for 4 h and cleared with 70% (*v*/*v*) ethanol. The GUS activity was determined using the GUS reporter gene quantitative analysis kit (Coolaber, Beijing, China).

### 4.6. Transient Transcription Activity Assay

To generate the constructs *pGmSPL9d ^H-I^*:LUC and *pGmSPL9d ^H-II^*:LUC, a 2000 bp promoter fragment was amplified from the *GmSPL9d* gene and ligated upstream of the pGreen0800-LUC reporter vector. These plasmids were then transformed into the GV3101 (P19) strain (Shanghai Weidi Biological). Subsequently, the plasmids were injected into *Nicotiana benthamiana* leaves. For the luciferase imaging, the Dual-luciferase assay reagent (Promega, Madison, WI, USA, VPE1910) was used, with Renilla luciferase serving as an internal control.

### 4.7. Arabidopsis Genetic Transformation

The constructs 35S: *GmSPL9d*, *pGmSPL9d ^H-I^*: *GmSPL9d*, and *pGmSPL9d ^H-II^*: *GmSPL9d* and the empty vector pCAMBIA1305 were individually transformed into *Agrobacterium* tumefaciens GV3101. These transformed bacteria were subsequently used for the floral dip transformation of *Arabidopsis* to generate transgenic plants [44].

### 4.8. Genetic Diversity Analysis

Variant data for *GmSPL9d* were retrieved from the public database SoyGVD (https://yanglab.hzau.edu.cn/SoyGVD/#/variation, accessed on 11 July 2023) and utilized for conducting genetic diversity analysis (Yang et al., 2023b).

### 4.9. Statistical Analysis

SPSS (https://www.ibm.com/spss, accessed on 11 July 2023) was employed to perform the statistical analysis. Student’s *t*-test or the Wilcoxon test were used to compare the differences between two groups, whereas Duncan’s multiple range test was used to compare the differences between multiple groups.

## Data Availability

All data supporting the findings of this study are available within the paper and within its supplementary data published online.

## Supplementary Materials

The following supporting information can be downloaded from https://www.mdpi.com/article/10.3390/ijms25115991/s1.
