# Supplementary figures and images for: Natural Variation in the Promoter of GmSPL9d Affects Branch Number in Soybean
Source: Int J Mol Sci. 2024 May 30;25(11):5991. doi: 10.3390/ijms25115991 (PMC11172651; doi:10.3390/ijms25115991)

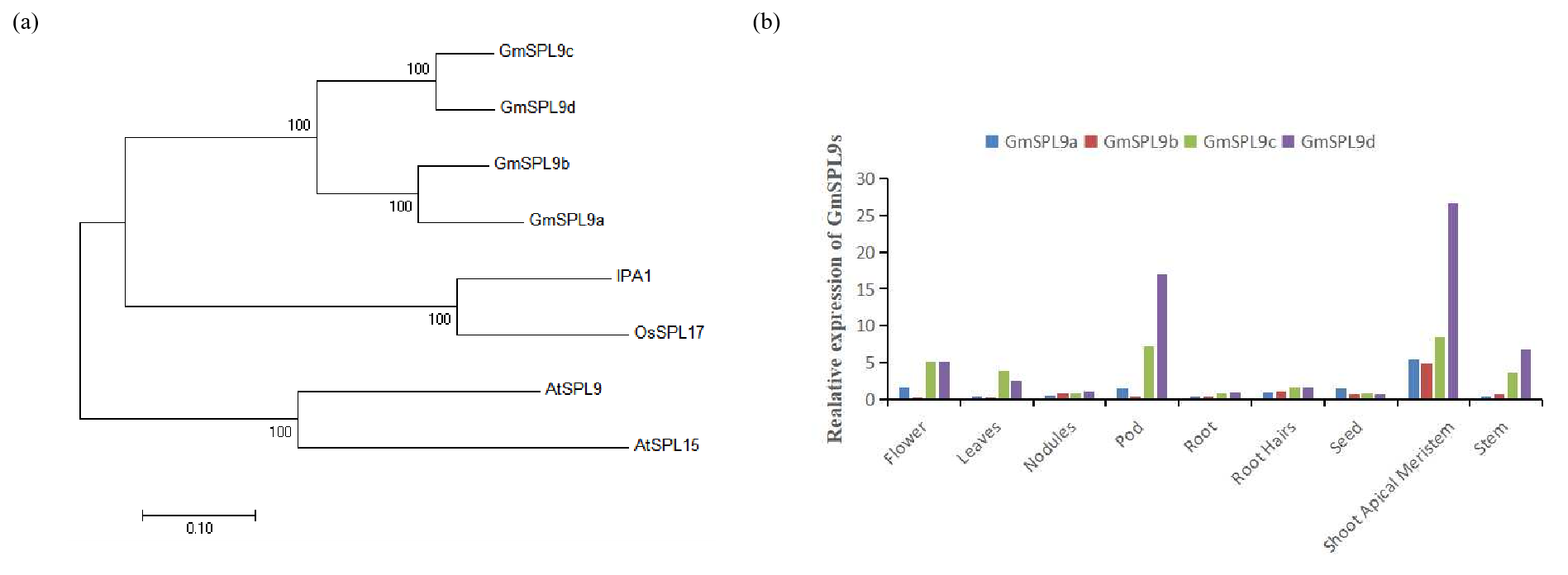

Supplement: Supplementary file 1 [file ijms-25-05991-s001.zip › Figure S1.tif]

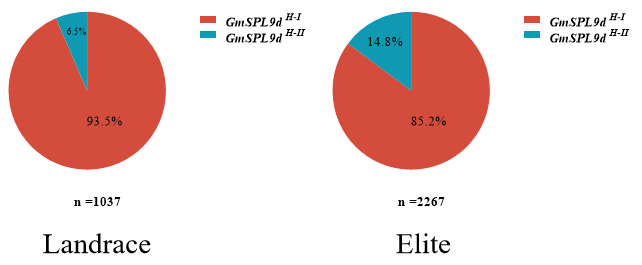

Supplement: Supplementary file 1 [file ijms-25-05991-s001.zip › Figure S2.tif]

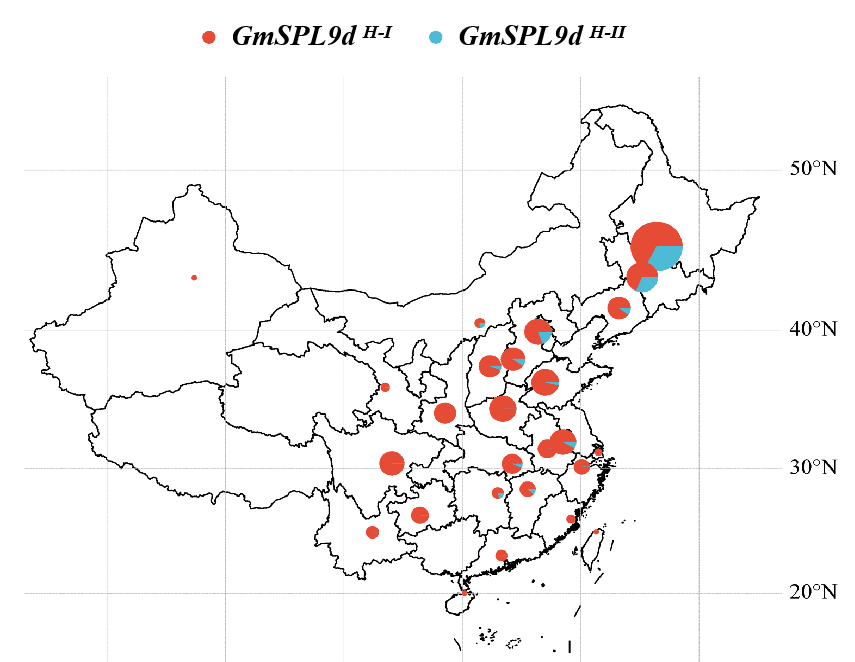

Supplement: Supplementary file 1 [file ijms-25-05991-s001.zip › Figure S3.tif]

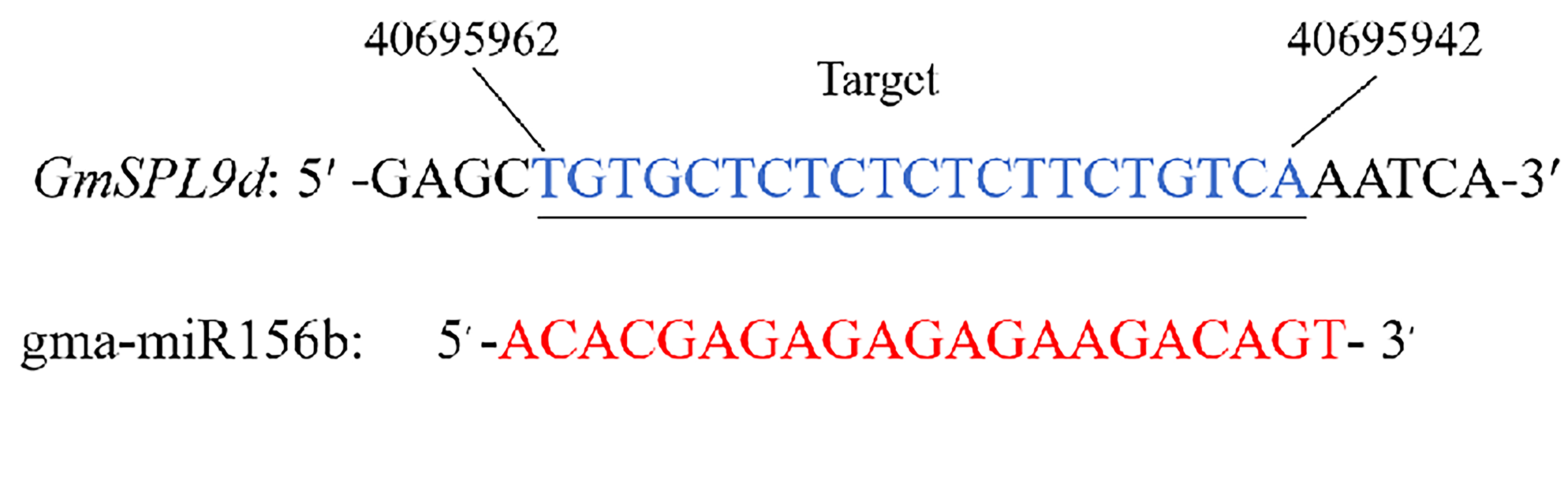

Supplement: Supplementary file 1 [file ijms-25-05991-s001.zip › Figure S4.tif]

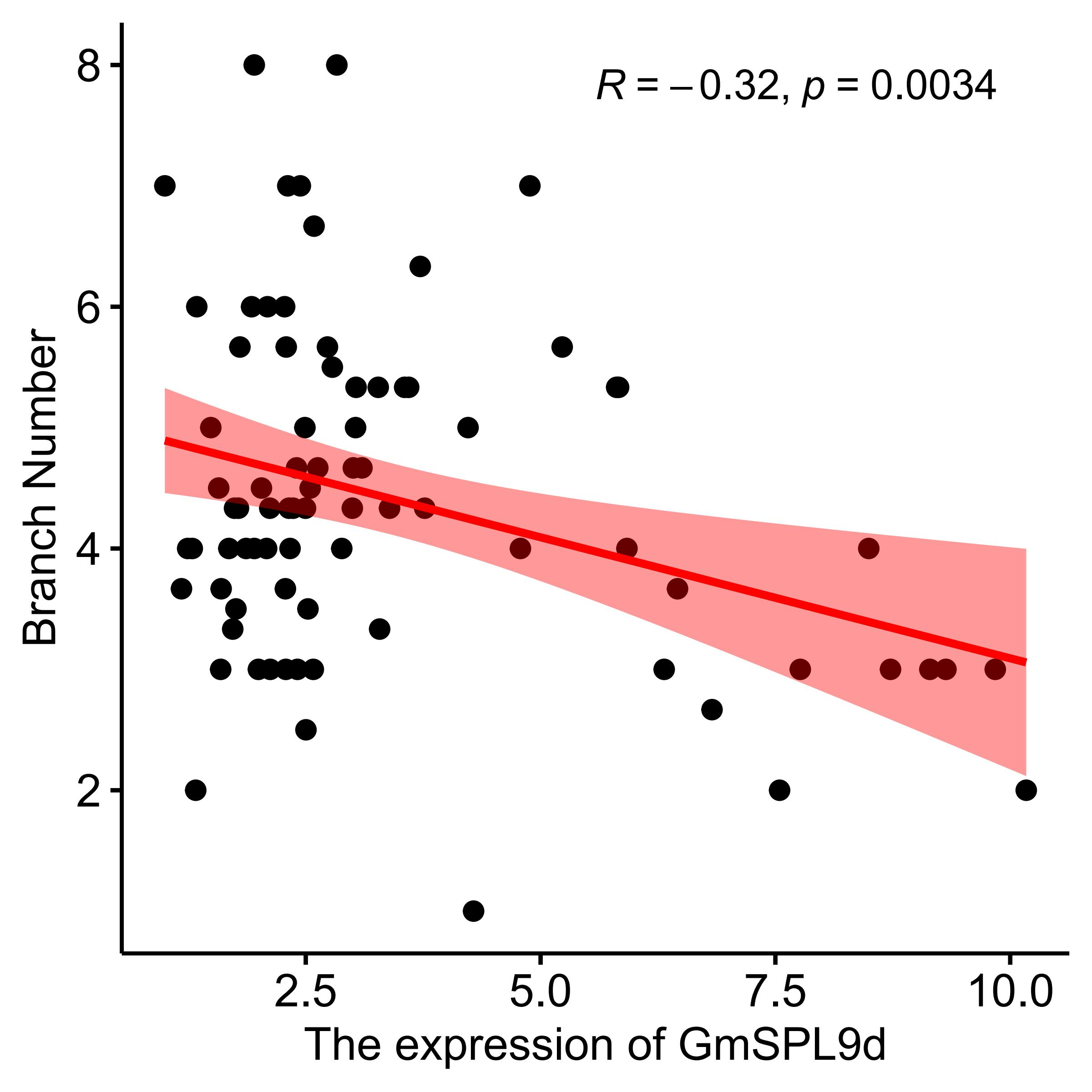

Supplement: Supplementary file 1 [file ijms-25-05991-s001.zip › Figure S5.tif]

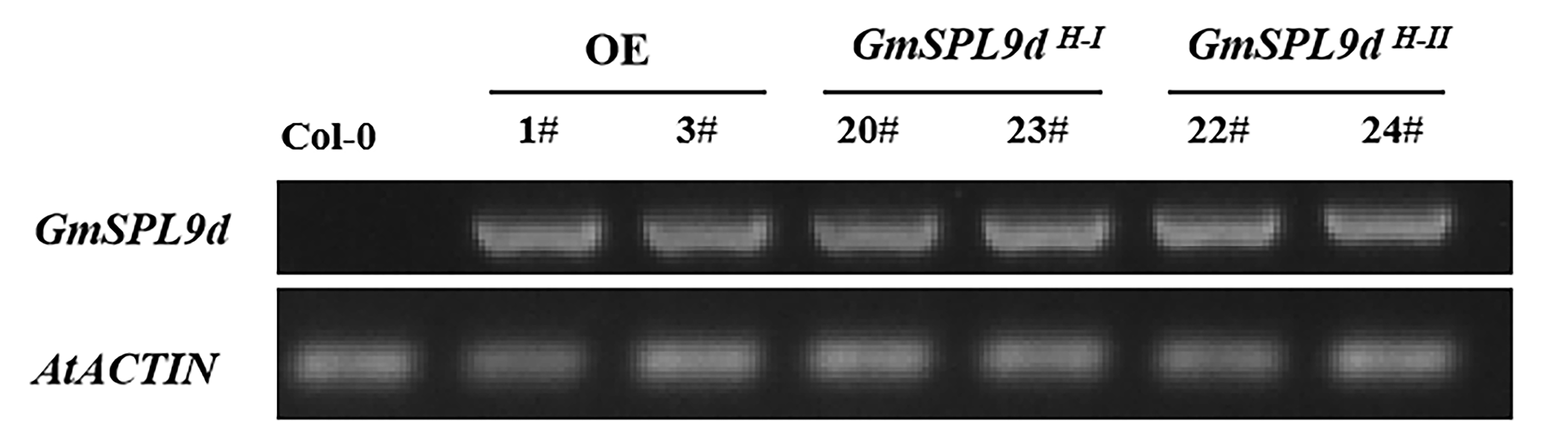

Supplement: Supplementary file 1 [file ijms-25-05991-s001.zip › Figure S6.tif]
